# Correlation of zero echo time functional MRI with neuronal activity in rats

**DOI:** 10.1177/0271678X251314682

**Published:** 2025-01-23

**Authors:** Juha S Valjakka, Jaakko Paasonen, Raimo A Salo, Ekaterina Paasonen, Petteri Stenroos, Irina Gureviciene, Mikko Kettunen, Djaudat Idiyatullin, Heikki Tanila, Shalom Michaeli, Silvia Mangia, Olli Gröhn

**Affiliations:** 1 A. I. Virtanen Institute for Molecular Sciences, University of Eastern Finland, Kuopio, Finland; 2 Center for Magnetic Resonance Research, University of Minnesota, Minneapolis, USA; 3 Neurocenter, Kuopio University Hospital, Kuopio, Finland

**Keywords:** Functional magnetic resonance imaging, zero-TE, ZTE, zero echo time, MB-SWIFT, hemodynamic, impulse response function, neurovascular coupling

## Abstract

Zero echo time (zero-TE) pulse sequences provide a quiet and artifact-free alternative to conventional functional magnetic resonance imaging (fMRI) pulse sequences. The fast readouts (<1 ms) utilized in zero-TE fMRI produce an image contrast with negligible contributions from blood oxygenation level-dependent (BOLD) mechanisms, yet the zero-TE contrast is highly sensitive to brain function. However, the precise relationship between the zero-TE contrast and neuronal activity has not been determined. Therefore, we aimed to derive a function to model the temporal dynamics of the zero-TE fMRI signal in response to neuronal activity. Furthermore, we examined the correlation of zero-TE fMRI with neuronal activity across stimulation frequencies. To these ends, we performed simultaneous electrophysiological recordings and zero-TE fMRI in rats subjected to whisker stimulation. The presented impulse response function provides a basis for the statistical modeling of neuronal activity-induced changes in the zero-TE fMRI signal. The temporal characteristics of the zero-TE fMRI response were found to be consistent with the previously postulated non-BOLD hemodynamic origin of the functional contrast. The zero-TE fMRI signal was well predicted by electrophysiological recordings, although systematic stimulation-dependent residuals were also observed, suggesting nonlinearities in neurovascular coupling. We conclude that zero-TE fMRI provides a robust proxy for neuronal activity.

## Introduction

Functional magnetic resonance imaging (fMRI) is the primary method for non-invasive mapping of brain function. The most common fMRI approach relies on the blood oxygenation level-dependent (BOLD) contrast,^1^ which originates from deoxyhemoglobin’s effect on the T_2_/T_2_*-relaxation of blood and the surrounding tissue.^2^ BOLD fMRI is conventionally performed using some variant of gradient- or spin-echo echo planar imaging (EPI), with an echo time (TE) in the order of tens of ms. While the BOLD contrast is exceptionally sensitive to brain function, EPI is disadvantaged by image artifacts related to magnetic susceptibility and motion, along with loud acoustic noise. The drawbacks of EPI limit the flexibility of fMRI study designs, and evidently, alternative approaches should be developed to fully realize the potential of fMRI in probing brain function in both clinical and preclinical settings.

Recently, an MRI sequence entitled multi-band sweep imaging with Fourier transformation (MB-SWIFT) has been adopted for functional imaging.^3^ MB-SWIFT belongs to a pulse sequence class known as zero echo time (zero-TE) MRI, which differs markedly from EPI. Namely, instead of sampling echo signals during Cartesian readouts, zero-TE MRI samples free induction decays during rapid radial readouts (TE <1 ms), thus providing a high acquisition bandwidth in three dimensions with minimal gradient switching between successive acquisitions.^4^ As a result, zero-TE MRI is fast and quiet, resilient to motion artifacts, and practically immune to image distortions caused by magnetic susceptibility gradients.^3–8^ Consequently, zero-TE fMRI has found applications in experimental settings that directly benefit from these features, such as deep brain stimulation fMRI,^3^ spinal cord fMRI,^9^ and behavioral fMRI in awake rats.^10^

Notably, as the zero-TE contrast has a negligible contribution from transverse relaxation,^4^ zero-TE sequences are not sensitive to the T_2_/T_2_*-dependent BOLD effect. Nonetheless, they function remarkably well for fMRI.^3,8,11^ Since zero-TE fMRI responses in the brain and spinal cord are suppressed when saturation bands are used to nullify the signal of in-flowing blood,^3,9^ a non-BOLD hemodynamic functional contrast has been assumed.

Hemodynamic signals are thought to correlate well with neuronal activity, but nonlinearities have also been reported.^12^ Since the origin of the functional contrast in zero-TE fMRI has not been fully characterized, it still remains an open question how the zero-TE functional signal reflects neuronal activity. In this work, we sought to examine this question by performing simultaneous zero-TE fMRI and intracranial electroencephalography (iEEG) during somatosensory-driven activity in the neocortex of isoflurane-medetomidine anesthetized rats. Specifically, our aims were to i) derive an impulse response function (IRF) for zero-TE fMRI and ii) explore the correlation of zero-TE fMRI with neuronal activity across varying stimulation conditions.

## Material and methods

### Animals

All animal procedures were approved by the Animal Experiment Board in Finland and conducted in accordance with Directive 2010/63/EU of the European Parliament and the ARRIVE guidelines. Nine male and eight female Sprague-Dawley rats were used (n = 17, age: 21 ± 7 weeks, male weight: 415 ± 54 g, female weight: 288 ± 34 g, [mean ± standard deviation]). Food and water were available *ad libitum*. All experiments were conducted between 8 a.m. and 5 p.m.

### Surgery

All rats underwent surgery to allow the placement of iEEG electrodes and a head-fixation implant under isoflurane anesthesia (5% induction, 2% maintenance, in a 30/70% 0_2_/N_2_ carrier gas, Attane vet 1000 mg/g, Piramal Healthcare UK Limited, Northumberland, UK). Four tungsten wire electrodes (⌀ 127 µm, Bilaney Consultants GmbH, Düsseldorf, Germany) were placed on dura via burr holes above the primary somatosensory cortex barrel field (S1BF). The coordinates of the four burr holes were −2.3, +5.5; −2.3, −5.5; −4.5, +6.0, and −4.5, −6.0 (mm from bregma, mm from midline), corresponding to bilateral locations in the middle and just posterior to S1BF.^13^ These coordinates were chosen based on their sensitivity to whisker-evoked potentials in pilot subjects. In addition to the tungsten wires, two skull screws (brass, F Reyher Nchfg GmbH & Co. KG, Hamburg, Germany) were placed in burr holes over the olfactory bulb, and another two over the cerebellum to serve as ground (GND) and reference (REF) electrodes. The head-fixation implant was implemented as previously described^8^ with additional anchoring provided by the brass screws. Access to the iEEG electrodes was provided via a custom-made connector plug on top of the implant (Supplementary Figure S1). Buprenorphine (0.03 mg/kg SC, Temgesic, Indivior Europe Ltd, Dublin, Ireland), carprofen (5 mg/kg SC, Rimadyl, Zoetis Belgium SA, Louvain-la-Neuve, Belgium) and saline (10 ml/kg/day SC) were given twice a day for two days for analgesia and rehydration.

### Whisker stimulation

Electrical stimulation of the whisker pad or air puff-based deflections of the whiskers were used to elicit activity in the rat somatosensory cortex. Both forms of stimulation were performed unilaterally and implemented with a general-purpose stimulus generator (STG4008-16mA, Multi Channel Systems MCS GmbH, Reutlingen, Germany) and the manufacturer’s software (MC_Stimulus II, V3.5.11). The side of stimulation was determined at the beginning of each imaging session based on a visual inspection of signal quality in the left and right iEEG channels, with the general aim being to produce equal amounts of data for both hemispheres.

For electrical stimulation, two electrodes built from 30 G stainless steel needles were placed between row-pairs A-B and C-D in the whisker pad such that most of their length (13 mm) was subcutaneous (Supplementary Figure S1). The leads from the needle electrodes were connected to the positive and negative poles of the stimulus generator, and the current was driven between them in biphasic pulses consisting of 2 mA for 300 µs followed by −2 mA for 300 µs.

In the air puff-based stimulation paradigm, posterior-to-anterior deflections were achieved with puffs of air directed at a piece of adhesive tape on the whiskers (Supplementary Figure S1). The tape on the whiskers was approximately 20 × 15 mm in size and attached to a maximal tuft of whiskers each time. The air puffs were produced with pressurized air (1–1.5 bar) gated by a solenoid valve (Neos Biotec, Pamplona, Spain) that opened in pulses of 5 ms in response to signals from the stimulus generator. The air was directed to the posterior side of the whiskers via 6 m of pneumatic pipe (6-mm ID) and 45 cm of flexible tube (3-mm ID) with an open end. The angle of the tube was fine-tuned for each experiment separately in order to achieve a clear and reliable deflection of the whiskers (20–40°). The reliability of the approach at high stimulation frequencies (up to 20 Hz) was confirmed by video recordings in pilot experiments (Supplementary Video S1).

### Magnetic resonance imaging

All MRI was performed with a 9.4 T/31-cm magnet interfaced with an Agilent DirectDrive console using a surface transceiver RF-coil (Neos Biotec, Pamplona, Spain) with an inner diameter of 22 mm, as previously described (Supplementary Figure S1).^8^ The RF excitation power was calibrated in a 10-mm horizontal slab with the upper edge at the surface of the cortex. The flip angles used in zero-TE MRI were between 4.5–6°.

Anatomical images were acquired using the MB-SWIFT pulse sequence^7^ with the following parameters: spoke TR ∼3 ms, 4 sub-pulses per spoke, excitation/acquisition bandwidth 192/384 kHz, matrix size 256 × 256 × 256, field-of-view 40 × 40 × 40 mm. Data was acquired using 16 segmented 4000-spoke groups, each traveling in a different pole-to-pole spherical spiral trajectory. As a way of enhancing the anatomical contrast, a magnetization transfer pulse (γB_1_ = 125 Hz, offset 2000 Hz, duration 20 ms) was employed every 32 spokes.

Zero-TE fMRI was performed using MB-SWIFT with the parameters set as follows: spoke TR 0.97 ms, 4 sub-pulses per spoke, excitation/acquisition bandwidth 192/384 kHz, matrix size 64 × 64 × 64, and field-of-view 40 × 40 × 40 mm. The number of volumes and the acquisition trajectory depended on the experimental aim (see Experimental overview). In Group 1 experiments, EVER-SWIFT^14^ was performed using a modified spoke trajectory (Supplementary Figure S2). EVER-SWIFT is an advanced zero-TE fMRI approach that allows the enhancement of temporal resolution via reordering of radial acquisitions collected during recurring events. In each Group 1 experiment, a time course of 140 EVER-SWIFT volumes with 2000 spokes per volume was obtained by combining data across 20 stimulation events, allowing a temporal resolution of ∼100 ms. In Group 2, conventional zero-TE fMRI experiments were performed; in each experiment, 930 volumes were acquired with 2047 spokes per volume and a pole-to-pole spherical spiral trajectory, leading to a temporal resolution of 2 s.

### Electrical recordings

Electrophysiological recordings were performed with MRI-compatible equipment (BrainAmp MR, Brain Vision LLC, Garner, NC, US) at a sampling rate of 5 kHz. The four intracranial tungsten electrodes and four skull screws were connected to an amplifier via eight individual leads twisted together to minimize electromagnetic interference from the gradient coils. Monopolar recording was done via the four tungsten electrodes using the screws above the left and right cerebellar hemisphere as GND and REF. If there were any signal quality issues attributable to GND or REF, the screws above the olfactory bulb were used as GND and REF instead. The electrophysiology system also recorded the trigger signals from the stimulation and MRI systems to mark the timings of stimuli and fMRI volumes, which were used in the iEEG data processing.

### Experimental overview

In all experiments, rats were anesthetized using a combination of isoflurane (Attane vet 1000 mg/g, Piramal Healthcare UK Limited, Northumberland, UK) and medetomidine (Domitor vet 1 mg/ml, Orion Corporation, Espoo, Finland). At the beginning of each session, anesthesia was induced with 5% isoflurane (in 30/70% 0_2_/N_2_ carrier gas) and the rat was placed in a custom-built MRI holder (polyoxymethylene), where isoflurane delivery was continued at 2% for the duration of set-up preparation. The preparation included fixing the rat’s head to the holder via the head implant (Supplementary Figure S1), ensuring the proper functioning of the electrical recording and whisker stimulation, and the placement of an infusion needle under the back skin. A medetomidine bolus (0.015 mg/kg SC) was given typically within 20 min from the onset of anesthesia, followed by a continuous infusion (0.03 mg/kg/h SC) starting 15 min later. As medetomidine was introduced, isoflurane was gradually reduced to 0.2–0.9%. The level of isoflurane was adjusted for each experiment separately with the goal being to achieve a steady respiration rate of 45–65 breaths per minute. A warm water circulation system (Corio CD, Julabo, Seelbach, Germany) was used to maintain the rat’s body temperature at 36–38°C and its respiration rate and body temperature were monitored throughout the experiments with dedicated equipment (Model 1025, Small Animal Instruments Inc., New York, NY, USA). When the experiments were terminated, the rats were injected with atipamezole (0.5 mg/kg SC, Antisedan vet 5 mg/ml, Orion Corporation, Espoo, Finland) and returned to their cages.

The experiments were performed in two separate groups with aim-specific study designs:

**Group 1**—Deriving the impulse response function (n = 4, 2 males, 2 females). In this case, electrical whisker pad stimulation was used. Stimulation consisted of 7 consecutive biphasic electric pulses at ∼0.143-s intervals (∼1 s of 7 Hz stimulation). In each experiment, the stimulation was delivered 20 times, with ∼47-s breaks, i.e. the duration of the experiments was 16 min (20 × [1-s stimulus + 47-s break]). In order to produce a single fMRI time course with enhanced temporal resolution using data from the 20 repeated stimuli, the exact timings of the stimuli were varied relative to data acquisition (Supplementary Figure S3). Each rat underwent three imaging sessions on separate days, when three such experiments took place.

**Group 2**—Investigating the correlation with neuronal activity (n = 13, 7 males, 6 females). In this set-up, both electrical whisker pad stimulation and air puff-based whisker stimulation were used. All of the stimulations were delivered in 16-s blocks of continuous stimulation, interposed with 44-s breaks. Stimulation was arranged with 7 different frequencies, defined as the number of biphasic electric pulses or air puffs delivered per second. Frequencies of 1, 3, 5, 7, 9, 13, and 17 Hz were used because based on previous studies, this range was deemed suitable for obtaining a variable response size.^15,16^ In each imaging session, two consecutive experiments took place: one with electrical stimulation and another with air puff stimulation with the same stimulation side being used in both experiments. In each experiment, 4 blocks of stimulation were delivered per stimulation frequency, leading to a total of 28 blocks of stimulus which meant that the duration of the experiment was 31-min (180-s baseline + 28 × [16-s stimulation + 44-s break]). The order of frequencies was randomized for each experiment separately, as was the order of electrical stimulation and air puff stimulation experiments in each session. Each rat underwent 1–3 imaging sessions on separate days.

### Data processing and analysis

Custom Python 3.10 and MATLAB (R2022b) scripts were used for all data processing and analysis.

An in-house Snakemake^17^ (https://snakemake.readthedocs.io/) script was used to perform the pre-processing steps of reconstruction of images into NIfTI and BIDS structure^18^ (https://bids.neuroimaging.io), co-registration, and motion correction.

The Agilent MRI data was reconstructed using in-house Python scripts for MB-SWIFT. The data was first Fourier-transformed from the k-space domain and correlated with the RF-pulse function. Next, the data was transformed back to the k-space domain and chopped to obtain the radial spokes. The data was then processed using re-gridding and an iterative FISTA^19^ algorithm where the reconstruction was performed volume-by-volume with 13 iterations.

In the experiments where left-side stimulation was used, anatomical and fMRI volumes were flipped horizontally. The reconstructed anatomical brain volumes were co-registered to a study-specific template using N4 bias correction,^20^ and linear and non-linear SyN^21^ registrations were performed with advanced normalization tools (ANTs) (http://stnava.github.io/ANTs).

As the contrast in the fMRI volumes was different from the usual EPI data, the series were motion-corrected with a tailor-made Python script using masking, N4 bias correction,^20^ and ANTs rigid co-registration of each volume to the first volume of the series. The motion-corrected fMRI series were moved to the template reference frame to allow easier group analyses using linear and non-linear transformations from the anatomical co-registration.

All of the fMRI time courses analyzed and presented in this work represent signals from a region of interest (ROI) drawn based on the anatomy of the S1BF in the left hemisphere.^13^ The ROI was first drawn on an anatomical template image, and then interpolated in the template fMRI reference frame to allow time course extraction. The fMRI response time courses were computed for each individual response as the average signal from within the ROI, and the signal was high-pass filtered using 100-s cutoff.

All iEEG data was bandpass filtered at 4–190 Hz, followed by notch filters at 50, 100 and 150 Hz to eliminate power-line noise. In each experiment, one of the two electrodes above the contralateral S1BF was chosen for analysis based on its sensitivity to stimulus-related activity; the mean power of the filtered iEEG signal during stimulation relative to the mean power during the whole experiment was calculated for both electrodes, and then the electrode with the higher value was used in the analysis.

The group-specific processing and analysis are described below.

**Group 1**—Before re-gridding and image reconstruction, spokes were reordered as illustrated in Supplementary Figure S3. The resulting 100-ms resolution fMRI time courses, each covering a 14-s window beginning 3 s before stimulus onset, were used to calculate a mean fMRI response time course to the 1-s whisker stimulation. Similarly, a mean iEEG response time course was calculated across all stimuli. The mean iEEG response time course was squared and baseline-corrected by subtracting the mean of the 3-s pre-stimulus baseline. A model function and a parameter value range were decided based on an initial visual optimization. The mean iEEG response was then convolved with the model function with varying combinations of parameter values and each convolution was fitted to the mean fMRI response. Before fitting, the convolved iEEG time course was temporally down-sampled by averaging over adjacent samples in 100-ms bins. The IRF was defined as the function with the combination of parameter values that led to the convolution with the smallest sum of squared residuals when fitted to the zero-TE fMRI response.

**Group 2**—Although MRI-related iEEG artifacts in Group 1 were efficiently removed by time-domain averaging, in Group 2, IRF-convolution was used on the single-trial recordings to avoid the unwarranted exclusion of non-phase-locked components of the iEEG response.^22^ Hence, each iEEG recording underwent MRI artifact removal. Because zero-TE fMRI causes only minor artifacts on simultaneously acquired electrical recordings,^8^ an artifact template subtraction method^23^ was deemed to be sufficient. An electrode-wise artifact template was calculated by averaging the iEEG signal over the timings of 90 fMRI volumes acquired during the 180-s baseline before the first stimulus block. This template was then subtracted from the iEEG signal throughout the recording prior to the application of bandpass and notch filters.

For each individual block of stimulation, fMRI and iEEG time courses were extracted using a 70-s time window beginning 28 s before stimulus onset. The iEEG time courses were squared, baseline-corrected by subtracting the mean of the 28-s pre-stimulus baseline, convolved with the IRF derived from Group 1, and temporally down-sampled by averaging over adjacent samples in 2-s bins.

The correlation of zero-TE fMRI with neuronal activity was assessed via linear regression. Linear models (*y* = *x*β_1_ + β_0_) were fitted between fMRI and neuronal response size. For fMRI, the response size was calculated as the sum of all time course values in a given response time window, z-score standardized from the responses in each experiment, and then averaged across all trials performed at each frequency in each subject. The neuronal response size was calculated in the same way from the IRF-convolved temporally down-sampled iEEG time courses. The correlation was also inspected by fitting mean IRF-convolved iEEG time courses to the mean fMRI time courses using a model without an intercept term (*y* = *x*β_1_). Mean time courses were calculated by first averaging across all trials in each subject, and then across subjects.

Statistical fMRI t-maps for each data set were computed using FLAME from FSL FEAT.^24^ At the individual experiment level, each stimulus was treated as being independent from the others. The group maps were produced by concatenating all of the experiment-level maps and testing if the response to either the electrical or air puff stimulus differed statistically from zero while taking multiple comparisons into account by using family-wise error (FWE) correction.

## Results

### Impulse response function for zero-TE fMRI (group 1)

Throughout the 12 imaging sessions (three per rat), a total of 36 experiments were conducted. No data needed to be discarded. This led to a total of 720 iEEG time courses and 36 fMRI time courses with 100-ms resolution after spoke reordering.

**Figure 1.**
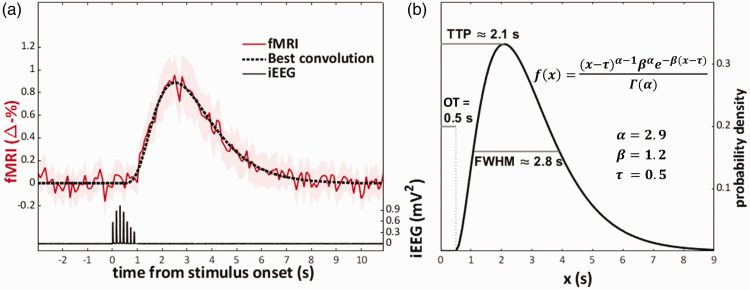
The zero-TE fMRI IRF derived from the rat somatosensory cortex. Data presented is from 720 stimulations conducted in four rats. (a) shows the fMRI response (mean of 36, 95% confidence interval shaded) and the neuronal response (mean of 720), along with a convolution of the neuronal response that best fitted the fMRI response and (b) shows the function and parameters for the best fitting convolution—a gamma probability density function supplemented with the parameter *τ* to quantify the response onset time. Abbreviations: full-width-at-half-maximum (FWHM), onset time (OT), time-to-peak (TTP).

Visual inspection of the mean zero-TE fMRI response was indicative of a shape consisting of a single positive curve (Figure 1(a)). Thus, the IRF was modeled as a gamma distribution function of the form

(1) $$f\left(x\right)=\{\begin{array}{ll} \frac{{\left(x-\tau \right)}^{\alpha -1}\beta^{\alpha }e^{-\beta \left(x-\tau \right)}}{\Gamma \left(\alpha \right)}, & x\geq \tau \\ 0, & x\mathrm{<\tau} \end{array}$$

where *Γ* denotes the gamma function, and *x* is time from stimulus onset in seconds. Optimization was done for the shape parameter *α* [0.1, 0.2 … 15], the rate parameter *β* [0.1, 0.2 … 15], and onset time *τ* [0, 0.1 … 1]. The best fit (R^2^ = 0.960) was achieved with *α = *2.9, *β = *1.2, and *τ = *0.5. Figure 1(b) shows the IRF along with the onset time (OT), and approximate values for time-to-peak (TTP), and full-width-at-half-maximum (FWHM).

### Correlation of zero-TE fMRI with neuronal activity (group 2)

Data from two sessions was excluded from the analysis due to complications related to the physiological state of the rat. In the remaining 26 sessions (1–3 per rat), 26 electrical stimulation experiments and 26 air puff stimulation experiments were carried out. Some data was further excluded as clearly artifactual; four fMRI time course points in one electrical stimulation experiment (>20 standard deviations from time course mean) and all iEEG time courses in one air puff experiment (persistent high amplitude external noise) were excluded, along with the iEEG and fMRI data measured in parallel. Hence, for the electrical and air puff stimulations, a total of 728 and 700 blocks of stimulation were included in the analysis, respectively (26 × 7 × 4 and 25 × 7 × 4 [experiments × stimulation frequencies ×blocks per stimulation frequency per experiment]).

Supplementary Figure S4 shows iEEG data from one representative fMRI session. For both electrical and air puff stimulation, iEEG responses were characterized by signs of increased power at the stimulation frequency and its harmonics (Supplementary Figure S4A,E). At stimulation frequencies above 1 Hz, the amplitude of responses during the 16 s of stimulation revealed clear evidence of adaptation, with the rate and degree of adaptation depending on the stimulation frequency (Supplementary Figure S4C,G). Responses to single stimuli (within a train of stimuli) displayed a shape consisting of an initial positive component, followed by a larger negative component, and ending in another slower positive component (Supplementary Figure S4D,H). These general observations were consistent across all experiments.

To illustrate the quality and contrast of anatomical and functional images, a comparison against fast spin-echo anatomical images is shown in Supplementary Figure S5. As seen from this figure, no major deterioration of signal quality around the tungsten electrodes was observed.

**Figure 2.**
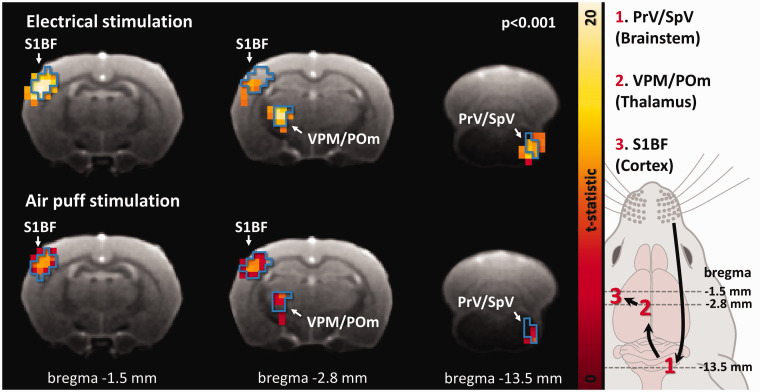
Group-level zero-TE fMRI activation maps from whisker stimulation overlaid on anatomical images. The maps were calculated using data from all 13 rats. In both electrical stimulation and air puff stimulation, activity was detected in regions corresponding to the lemniscal (PrV-VPM-S1BF) and paralemniscal (SpV-POm-S1BF) sensory pathways.^66^ The ROIs (blue borders) are based on an anatomical atlas.^13^ The two thalamic nuclei and the two brainstem nuclei were paired in the same ROIs due to their proximity. The p-value has been FWE-corrected. The schematic on the right illustrates the whisker pathway. The dashed lines in the schematic mark the approximate locations of the MRI slices shown on the left. Abbreviations: posterior thalamic nucleus (POm), principal trigeminal nucleus (PrV), spinal trigeminal nucleus (SpV), primary somatosensory cortex barrel field (S1BF), ventral posteromedial thalamic nucleus (VPM).

As expected, in both electrical and air puff stimulation, zero-TE fMRI revealed activity within the primary whisker pathway; highest t-values were located within the ROIs drawn based on the anatomy of the core structures (Figure 2). All key nodes of the whisker pathway were well visualized in the statistical maps, likely because all stimulation frequencies were included in the analysis and the stimulation frequency generating the biggest responses appears to be different for the cortex, thalamus, and the brain stem.^37^

**Figure 3.**
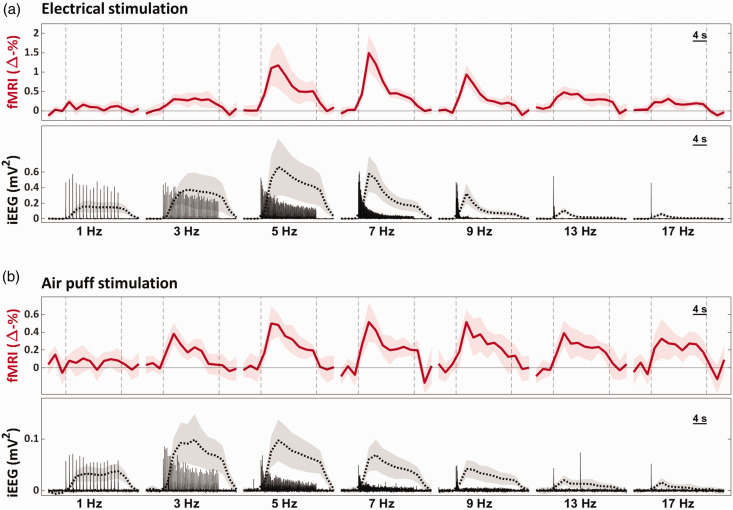
Zero-TE fMRI and neuronal response time courses at varying stimulation frequencies. Data presented is from 13 rats, from which a total of 104 and 100 blocks of stimulation (16 s) were included per frequency, for electrical (a) and air puff (b) stimulation, respectively. Each time course is a mean of means (a mean of 13 subject-wise mean time courses, each averaged over 4–12 time courses, depending on the number of experiments done in each subject). Shaded areas indicate 95% confidence intervals. Dashed vertical lines in the fMRI plots mark the onset and end of stimulation. For neuronal recordings, both plain time-averaged responses (solid line) and IRF-convolved, temporally down-sampled, and then time-averaged responses (dotted line) are shown. The scale in the iEEG plots (mV^2^) is for non-convolved responses; the unit of IRF-convolved responses is arbitrary.

With respect to electrical stimulation, fMRI response time courses showed a clear frequency-dependent variation in terms of both peak amplitude and shape (Figure 3(a)). A similar frequency-dependent variation was observed in the IRF-convolved iEEG recordings. In both measures, responses increased from 1 Hz to 5–7 Hz and decreased thereafter. When we conducted the air puff stimulation, the responses were notably smaller in both fMRI and iEEG (Figure 3(b)). Although neuronal responses to air puff stimulation exhibited a frequency-dependent variation similar to that seen in electrical stimulation, the variation in the fMRI responses was less apparent, decreasing only slightly after peaking at 5–7 Hz.

**Figure 4.**
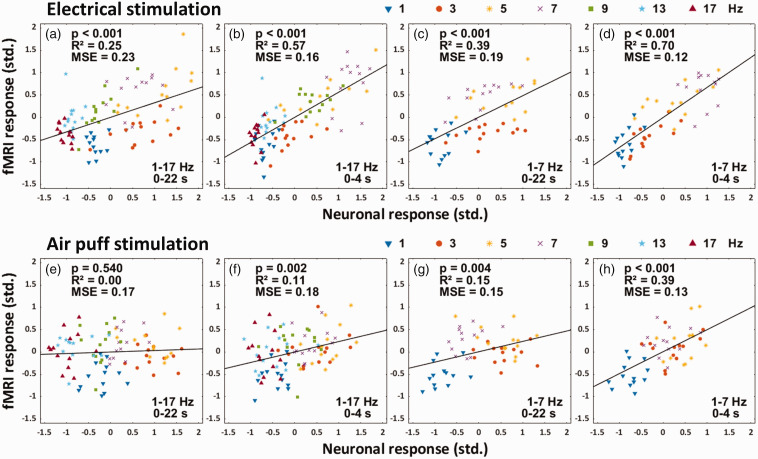
The correlation between zero-TE fMRI and neuronal response size. The response size was calculated from a time window representing either the full 22 s of the response (a,e), or the first 4 s (b,f). The correlation was also assessed with the analysis restricted to stimulation frequencies 1–7 Hz, again in time windows representing either the full 22 s (c,g), or the first 4 s (d,h). Response size refers to the sum of time course values in a given time window. In every plot, each data point represents the mean response in one subject at one stimulation frequency (averaged over 4–12 responses, depending on the number of experiments done in each subject). Before averaging, responses in each experiment were z-score standardized. Abbreviations: mean-squared-error (MSE), standardized (std.).

Figure 4 shows the correlation between neuronal and zero-TE fMRI response size. Based on the expected lag of the response (Figure 1(b)), the response size was calculated using a 22-s time window beginning at stimulation onset and ending 6 s after the stimulation. In electrical stimulation, neuronal responses linearly explained approximately 25% of the variation in zero-TE fMRI responses (Figure 4(a)). Responses at each frequency appeared to cluster either below or above the regression line, suggesting the presence of frequency-related nonlinearity between the two measures. If the response size was calculated from a time window that represented the first 4 s of stimulation, then the responses distributed more centrally around the regression line, and the fit of the linear model was improved, as reflected in the increased coefficient of determination (R^2^) and decreased mean-squared-error (MSE) (Figure 4(b)). A better fit was also achieved if the analysis was restricted to frequencies 1, 3, 5 and 7 Hz (Figure 4(c)). An even better fit was achieved when analysis was restricted to both the first 4 s of stimulation and frequencies 1–7 Hz, with neuronal responses explaining 70% of variation in zero-TE fMRI responses (Figure 4(d)). Similar conclusions could be drawn from air puff stimulation experiments; while neuronal responses did not seem to explain the zero-TE fMRI responses when all data was considered (Figure 4(e)), 39% of variation between the two measures was explained during the first 4 s of stimulation at frequencies 1–7 Hz (Figure 4(h)).

**Figure 5.**
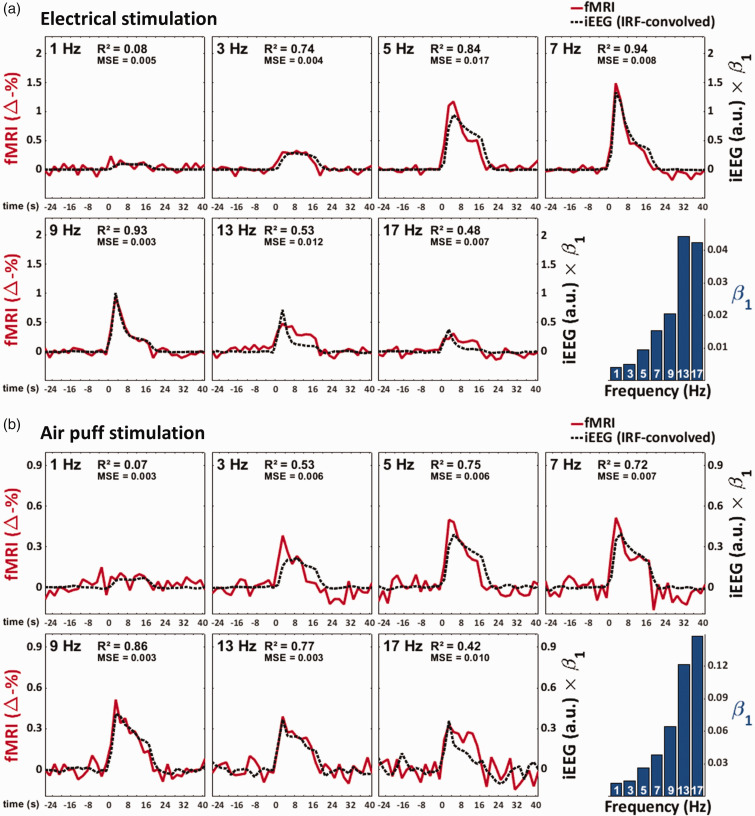
The correlation of zero-TE fMRI response time courses with IRF-convolved neuronal recordings. (a) and (b) show the responses to electrical and air puff stimulation, respectively. As in Figure 3, each time course is a mean of means, calculated from 13 rats. Here, IRF-convolved neuronal recordings have been fitted to the fMRI response time courses separately for each stimulation frequency. Hence, although the scale of fMRI is constant across plots, the scale of the IRF-convolved neuronal response varies by plot, in accordance with the frequency-wise regression coefficients (β_1_) shown in the bar charts. In the time course plots, the x-axis shows the time from stimulus onset. Abbreviations: arbitrary units (a.u.), mean-squared-error (MSE).

To inspect the correlation of zero-TE fMRI with neuronal activity with more temporal detail, IRF-convolved iEEG time courses were fitted to the fMRI time courses with the fits being done separately for each frequency (Figure 5). In most cases, the IRF-convolved iEEG response predicted well the temporal profile of the fMRI response. However, in both electrical and air puff stimulation, the regression coefficient (β_1_) increased with stimulation frequency. Furthermore, even with frequency-wise regression coefficients, there seemed to be a general trend for the neuronal response to either under- or overestimate the peak-to-plateau ratio of the fMRI response, at the lower and higher ends of frequencies, respectively.

The above-mentioned discrepancies between the fMRI and iEEG responses are visualized in more detail in Supplementary Figure S6. In this figure, we used the same scaling for all iEEG time courses, derived by calculating a regression coefficient for the first 4 s of the 7 Hz response time course. The beginning of the 7 Hz stimulation corresponds to the stimulation condition under which the IRF was derived; therefore, an IRF-convolution of the iEEG response should fit to the fMRI response with minimal residuals (Figure 1(a)). Residuals could arise at other stimulation time points and frequencies, however, due to stimulation-dependent nonlinearities. As shown in Supplementary Figure S6, such nonlinearities were present across all stimulation time points and frequencies.

## Discussion

In this work, we investigated the zero-TE fMRI response at 100-ms temporal resolution, allowing us to reliably model the onset time (OT), time-to-peak (TTP), and full-width-at-half-maximum (FWHM) of the response, and derive an IRF for use in statistical modeling. As discussed below, the temporal characteristics of the zero-TE fMRI response closely resembled those of hemodynamic responses, particularly cerebral blood volume (CBV) and cerebral blood flow (CBF).

Furthermore, we have provided evidence that there is a correlation between the zero-TE fMRI signal and neuronal activity by demonstrating that its amplitude and temporal dynamics were adequately predicted by neuronal recordings. However, linear fits between neuronal and fMRI responses also had consistent stimulation-dependent residuals which could be related to either inherent nonlinearities in neurovascular coupling,^25,26^ or biases of epidural iEEG as a measure of neuronal activity.

### The zero-TE fMRI impulse response function

Previously the temporal features of hemodynamic impulse responses to sensory stimulation have been characterized in the rat somatosensory cortex.^27–31^ The parameters investigated in these studies included CBV, CBF, and BOLD. Some researchers have determined TTPs, FWHMs, and OTs from responses to impulse-like stimuli without the need for modeling,^28–30^ while others have derived IRFs from longer stimuli (1–2 s) by assuming a constant neuronal response^31^ or recording it^27^ as was done here.

Table 1 lists TTP, FWHM and OT values from the current and previous studies, as either reported directly by the authors or determined from the presented IRF. It is clear that the values of the zero-TE fMRI IRF are similar to those previously reported for hemodynamic correlates of neuronal activity.

**Table 1.** TTP, FWHM and OT values from previous studies investigating hemodynamic responses in the rat somatosensory cortex.

Source	Measure	Technique	TTP (s)	FWHM (s)	OT (s)	Model function
Current study	?	MRI	2.1	2.8	0.5	$f\left(x\right)=({x-\tau )}^{\alpha -1}\beta^{\alpha }e^{-\beta \left(x-\tau \right)}{\Gamma \left(\alpha \right)}^{-1}$
Martindale et al.^27^	CBV	OIS	2.0	2.3	–	$f\left(x\right)=e^{-x\delta /\tau }x^{{(\delta }^{2}/x)-1}$
	CBF	LDF	2.1	1.9	–	
Silva et al.^28^	BOLD	MRI	2.2	1.9	–	–
	CBV	MRI	1.6	1.4	<0.5	–
Hirano et al.^29^	BOLD	MRI	1.6	1.5	0.4	–
	CBV	MRI	1.1	1.1	0.3	–
	CBF	MRI	1.1	1.0	0.4	–
Urban et al.^30^	CBV	fUS	0.8	1.5	0.4	–
Lambers et al.^31^	BOLD	MRI	3.1	2.1	–	$f\left(x\right)=e^{-bx}(b^{p_{1}}x^{p_{1}-1}{\Gamma \left(p_{1}\right)}^{-1}-b^{p_{2}}x^{p_{2}-1}{V^{-1}\Gamma (p_{2})}^{-1})$

In studies where a model function is missing, the authors have determined the values from responses to impulse-like stimuli. Neither Martindale et al. nor Lambers et al. modeled the OT, hence the missing values. Silva et al. stated that the CBV signal was above zero 0.5 s after stimulus onset but did not explicitly report the OT for BOLD. Hirano et al. reported separate values for different cortical layers, which were averaged for this table. The parameter values for the models derived by Martindale et al. and Lambers et al. are as follows: Martindale CBV: δ = 2.49, τ = 1.22; CBF: δ = 2.37, τ = 0.76; Lambers BOLD: b = 2.5, p1 = 10, p2 = 11.7, V = 1.5. Abbreviations: blood oxygenation level-dependent (BOLD), cerebral blood flow (CBF), cerebral blood volume (CBV), functional ultrasound (fUS), full-width-at-half-maximum (FWHM), laser Doppler flowmetry (LDF), magnetic resonance imaging (MRI), optical imaging spectroscopy (OIS), onset time (OT), time-to-peak (TTP).

**Figure 6.**
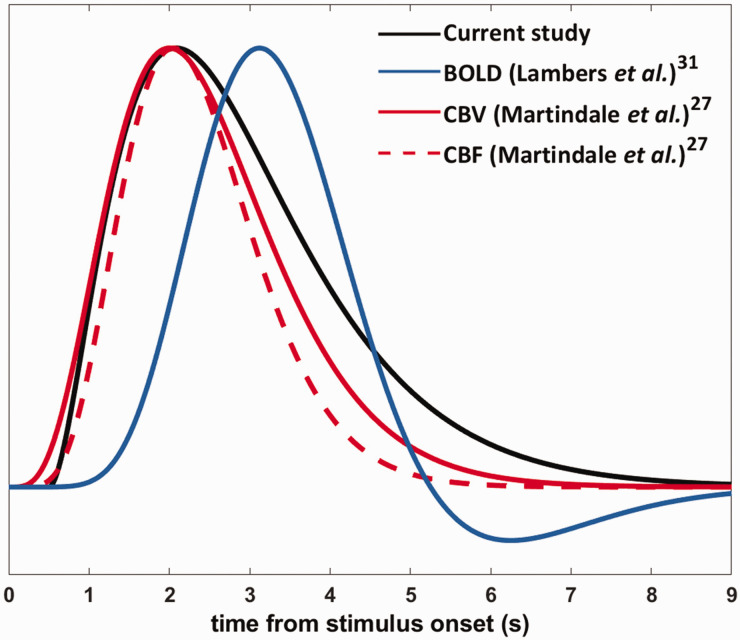
The zero-TE fMRI IRF along with hemodynamic IRFs derived from the rat somatosensory cortex in previous studies. See Table 1 for the model functions and parameters.

Figure 6 reveals that the IRF derived in the current study greatly resembles the CBV and CBF IRFs derived by Martindale et al. In comparison, the BOLD IRF derived by Lambers et al. was delayed, displayed a post-stimulus undershoot and a steeper post-peak slope. Although these (dis)similarities could be purely coincidental, they do agree with the previously hypothesized non-BOLD hemodynamic origin of the zero-TE fMRI signal.^3^ Specifically, local RF coil transmission during continuous inflow of blood would be expected to enhance the difference in the steady-state longitudinal magnetization (M_ss_) of blood and tissue (blood M_ss_ > tissue M_ss_), creating a contrast sensitive to both CBV (due to change in blood/tissue ratio) and CBF (due to the change in the inflow-dependent blood M_ss_). The concepts that the BOLD response should be delayed relative to CBV and CBF, and that the BOLD response would exhibit a post-stimulus undershoot missing in CBV and CBF responses (Figure 6), are supported by the results of Hirano et al.,^29^ who measured all three under consistent experimental conditions (Table 1).

It is important to note that the zero-TE fMRI IRF derived here was likely influenced by experimental conditions that affected the vascular dynamics. For example, anesthesia may lengthen the TTP and FWHM of hemodynamic IRFs,^16^ and it is reasonable to expect that this effect will further be influenced by the anesthetic agent and its dose. Notably, the rat BOLD IRF has been speculated to vary depending on brain region (cortical vs. subcortical), stimulation duration and mode (optogenetic vs. sensory), and respiration mode (spontaneous vs. ventilated).^31^ Differences in hemodynamic IRFs may also be evident between species. Thus, although there can be some limitations to the applicability of the zero-TE fMRI IRF presented here, it can be considered as a starting point for further studies characterizing the zero-TE fMRI IRF in different experimental conditions. Under the assumption that zero-TE fMRI is sensitive to a CBV/CBF-type contrast, a CBV/CBF IRF derived in any given conditions may adequately approximate the appropriate zero-TE fMRI IRF for those specific conditions.

### Correlation of zero-TE fMRI with neuronal activity

The premise underpinning the application of any MRI sequence for functional imaging is that it is sensitive to a contrast that correlates with neuronal activity. As zero-TE fMRI appears to be sensitive to CBV/CBF effects,^3,9^ which are established markers of neuronal activity, and sensory stimulation induced signal changes in the expected sensory structures (Figure 2), it is evident that the correlation of the zero-TE fMRI signal with neuronal activity should be sufficient to allow mapping of brain activity. Nonetheless, we wanted to conduct simultaneous electrophysiological recordings to evaluate the extent to which the zero-TE contrast reflects the amplitude and temporal dynamics of underlying neuronal activity. As a first approximation, we observed that the amplitude and temporal dynamics of zero-TE fMRI responses were well predicted by IRF-convolved neuronal recordings (Figure 3(a)). However, further inspection revealed a systematic residual component in the fMRI response that was not predicted by the neuronal response (Figure 3(b); Figure 4(a) and (e)).

Motivated by i) the fact that many previous studies on neurovascular coupling have utilized much shorter stimuli (1–4 s)^15,16,32,33^ and ii) the observation that fMRI and iEEG responses peaked around 7 Hz (Figure 3), we decided to inspect the fit of the linear model while only considering the first 4 s of stimulation, frequencies 1–7 Hz, or both (Figure 4(b) to (d), (f) to (h)). Under these confined conditions, linear fits to the data were notably better, suggesting that the residual component in the fMRI response is related to both the duration and frequency of the stimulation. Moreover, these residuals were more prominent in air puff stimulation than encountered with electrical stimulation (Figure 3; Figure 4(a) and (e)). Although this may be partially related to the fact that responses to air puff stimulation were generally smaller (lower signal-to-noise ratio), the frequency-modulation profiles of neuronal and fMRI responses did seem to dissociate more clearly in air puff stimulation than in electrical stimulation (Figure 3).

Next, we investigated temporal correlations by fitting the IRF-convolved neuronal response time courses to the fMRI response time courses (Figure 5). We found that while the time course fits were mostly good, the regression coefficients increased with stimulation frequency, and the peak-to-plateau ratios of neuronal and fMRI responses developed differently across the frequencies. As suggested by these results, we found that the residual component in the fMRI response changed throughout stimulation time points and frequencies (Supplementary Figure S6).

Because many investigations into neurovascular coupling in rats have used shorter stimulus lengths,^15,16,32,33^ they may have missed the nonlinearities related to longer stimuli. In those studies where longer stimulus lengths were used, stimulation duration-dependent nonlinearities were not explored to the same extent as here,^34–37^ except for a few studies.^25,26,38^ Kim et al.,^38^ who observed frequency-dependent BOLD and CBF responses similar to the current study, examined the coupling between neuronal and CBF responses in stimulation time windows of 0–10 s, 10–20 s, and 20–30 s.^38^ They concluded that the coupling remained linear in all stimulation time windows, seemingly contradicting with our observations. On the contrary, Ances et al.^25^ and Martindale et al.^26^ observed that a convolution of the neuronal response to 16 s of 5 Hz somatosensory stimulation mispredicts the peak-to-plateau ratio of the CBF response, in line with our observations (Figure 5). Both Ances et al.^25^ and Martindale et al.^26^ proposed that for stimuli lasting more than 4 s, a nonlinear convolution model would be required to accurately predict the time course of a hemodynamic response from neuronal recordings. This suggestion is consistent with our observation that the fit of the linear model was better when only data from the first 4 s of stimulation was being utilized (Figure 4). Why Kim et al.^38^ did not observe this nonlinearity is unclear, but it could be explained by differences in analysis or measurement techniques, or differences in experimental conditions, such as different anesthesia protocols.

Notably, our finding that the peak-to-plateau mismatch changed with stimulation frequency is an indication that even a nonlinear (time-variant) convolution model would not perform well across different stimulation frequencies (Figure 5). Thus, it seems likely that the type of neuronal recording performed here and in previous studies^25,26^ is simply blind to some type of activity that drives hemodynamics during sustained and high frequency stimulation.

### Epidural iEEG as a measure of neuronal activity

Regarding the correlation of zero-TE fMRI with neuronal activity, it should be noted that the definition of “neuronal activity” in the experimental sense depends on the measuring technique being used. In the current study, neuronal activity was defined as the voltage recorded via epidural electrodes placed above the somatosensory cortex, bandpass filtered between 4–190 Hz, and squared to obtain instantaneous power. In the cortex, electrophysiological signals in this frequency range are classically assumed to reflect postsynaptic potentials at the apical dendrites of pyramidal neurons.^39,40^ Although pyramidal neuron activity is thought to be the core driver of the sensory-driven hemodynamic response,^41,42^ other forms of cortical activity that may go undetected in such recordings can also contribute to the hemodynamic response. For instance, optogenetic activation of inhibitory interneurons^43–45^ and astrocytes^46^ has been claimed to evoke hemodynamic responses even in the absence of changes in the activity of the pyramidal neurons. Interestingly, both inhibitory interneurons^47^ and astrocytes^48^ have been proposed to participate in sensory adaptation, suggesting that they may be involved in regulating hemodynamics in times of longer stimuli, especially at high stimulation frequencies. Indeed, sensory-driven astrocytic activity exhibits a delayed component^49^ which is thought to contribute to hemodynamic responses during sustained stimulation.^50–52^ However, there is no clear evidence of the response patterns of pyramidal neurons and interneurons or astrocytes dissociating in a stimulation frequency dependent manner that would explain the systematic mispredictions seen in Figure 5 (although, see references^50,53^^–55^).

The above discussion might also explain why the frequency-modulation of fMRI responses was better predicted by iEEG in electrical stimulation than air puff stimulation (Figure 3); one could speculate that the activation of pyramidal neurons relative to other cell types is greater to electrical stimulation than to air puff stimulation.

The epidural iEEG approach used in this study may also be biased towards activity closer to the cortical surface because the voltage measurements are inversely associated with the distance from the point of measurement.^40^ Although the spatial reach of epidural iEEG is unclear, in comparison to intracortical microelectrode recordings, the approach used here would be expected to detect activity from a larger population of neurons due to the relatively large electrode size (⌀127 µm) and the greater distance to the individual neurons.^56^ Therefore, epidural recordings may capture best the total activity at the S1BF, the area over which the zero-TE fMRI signal was averaged when examining the correlation. However, adopting a higher spatial signal integration also has its disadvantages. Here, responses to individual stimuli (single electric pulse/air puff) consisted of positive and negative components (Supplementary Figure S4D,H), presumably arising from sequential activation of local neuronal populations within the volume recorded.^39^ The activation of these neuronal populations could display a temporal overlap not only during the response to a single stimulus, but also across consecutive stimuli, particularly at high stimulation frequencies. Should an overlap occur between populations that produce voltage deflections of opposite signs, signals from the volume recorded would cancel each other out, leading to a misrepresentation of the cortical response. Therefore, based on the current work, we cannot exclude the possibility that this bias may explain some of the deviation between iEEG and zero-TE fMRI at 13–17 Hz. Nonetheless, it is safe to assume that most of the frequency-dependent suppression in iEEG occurs due to a true adaptation of cortical neurons.^47^

Despite these biases, epidural iEEG is a commonly used measure of neuronal activity, and its sensitivity to pyramidal neuron activity was appropriate for the purpose of the current study, as this form of cortical activity is considered to be best linked to neurovascular coupling.^57,58^ While many studies on neurovascular coupling in rats have used intracortical electrodes,^15,16,32,35–38^ the epidural electrodes used in the current study have the benefit of avoiding insertion damage and the consequent gliosis and disturbance of tissue function.^59^

### Implications for fMRI research

Among the available fMRI techniques, zero-TE fMRI stands out in its quietness and striking insensitivity susceptibility-induced artifacts and body motion. These features owe to the characteristics of zero-TE radial acquisition: the minimal acquisition delay, the frequent sampling of the central k-space, the lack of low-bandwidth phase encoding, and the minimal gradient switching required for spatial encoding.^4^ Due to its insensitivity to susceptibility-induced artifacts, zero-TE fMRI is particularly suitable for imaging in the vicinity of brain implants^3^ and air cavities of the skull.^8^ Another emerging application of zero-TE fMRI is the imaging of awake animals,^8,10,60^ where motion artifacts and scanner noise-induced animal stress are major challenges.^61^

One further advantage of zero-TE fMRI exploited here is its suitability for simultaneous electrophysiological recordings.^8^ This advantage is twofold: i) the minimal TE and high acquisition bandwidth render the acquired images insensitive to distortions near the recording electrodes and ii) the small gradient coil steps minimize interference with electrical recordings; consequently, the quality of both functional images and the electrophysiology signal is maintained. In the current study, the ease of combining the two methods facilitated the validation of the zero-TE contrast as a correlate of neuronal activity, promoting the further adoption of zero-TE fMRI techniques for neuroscientific research.

Although zero-TE fMRI is a good general technique for quiet and artifact-free functional imaging, it is still unclear whether it is worth employing in experimental settings where field inhomogeneity, motion, or acoustic noise are not a major issue. This depends on how the zero-TE functional contrast compares to the standard BOLD contrast in two properties: sensitivity and spatial specificity. Regarding the latter, the IRF derived here may provide some insight; the zero-TE IRF indicates a CBV/CBF-type contrast (Figure 6), suggesting that the zero-TE fMRI signal localizes better to the site of neuronal activity than the BOLD fMRI signal. This notion is based on the observation that CBV/CBF contrasts are most sensitive to micro-vessels that reside at the site neuronal activity whereas the T_2_*-weighted BOLD contrast is sensitized to veins downstream from the site of activity.^62–64^ Although a micro-vascular BOLD contrast may be achieved via T_2_-weighting, this comes at the cost of reduced sensitivity.^65^ The sensitivity of the zero-TE functional contrast, in turn, is comparable to the T_2_-weighted BOLD contrast.^3^ Little work has been done for the optimization of imaging parameters and readouts in zero-TE fMRI, however. Thus, in terms of sensitivity, the full potential and limitations of zero-TE fMRI are yet to be demonstrated.

In this work, we also reported a residual component in the zero-TE fMRI response that was not predicted by IRF-convolved iEEG. Because the zero-TE functional signal originates mainly from changes in CBF and CBV, we expect that a similar residual component would have been observed with perfusion-based fMRI approaches and BOLD fMRI. Importantly, we observed that the residual component had a systematic dependence on the duration and frequency of stimulation, which may prove useful for future investigations into its origin. Such investigations are key to the correct interpretation of BOLD, CBV, and CBF as surrogate markers of brain activity.

### Conclusion

The IRF presented in this work provides a basis for the statistical modeling of neuronal activity-induced changes in the zero-TE fMRI signal in small rodents. The temporal characteristics of the zero-TE fMRI response were indicative of a CBV/CBF-type contrast. The simultaneous electrophysiological recordings showed that zero-TE fMRI provides a good proxy for neuronal activity, with some limitations, as expected for any measure that relies on neurovascular coupling. These findings are applicable to all zero-TE sequences.

## Supplemental Material

sj-pdf-1-jcb-10.1177_0271678X251314682 - Supplemental material for Correlation of zero echo time functional MRI with neuronal activity in rats Supplemental material, sj-pdf-1-jcb-10.1177_0271678X251314682 for Correlation of zero echo time functional MRI with neuronal activity in rats by Juha S Valjakka, Jaakko Paasonen, Raimo A Salo, Ekaterina Paasonen, Petteri Stenroos, Irina Gureviciene, Mikko Kettunen, Djaudat Idiyatullin, Heikki Tanila, Shalom Michaeli, Silvia Mangia and Olli Gröhn in Journal of Cerebral Blood Flow & Metabolism

sj-mp4-2-jcb-10.1177_0271678X251314682 - Supplemental material for Correlation of zero echo time functional MRI with neuronal activity in rats Supplemental material, sj-mp4-2-jcb-10.1177_0271678X251314682 for Correlation of zero echo time functional MRI with neuronal activity in rats by Juha S Valjakka, Jaakko Paasonen, Raimo A Salo, Ekaterina Paasonen, Petteri Stenroos, Irina Gureviciene, Mikko Kettunen, Djaudat Idiyatullin, Heikki Tanila, Shalom Michaeli, Silvia Mangia and Olli Gröhn in Journal of Cerebral Blood Flow & Metabolism
