# Supplementary material for: Correlation of zero echo time functional MRI with neuronal activity in rats
Source: J Cereb Blood Flow Metab. 2025 Jan 23;45(5):855–70. doi: 10.1177/0271678X251314682 (PMC11758440; doi:10.1177/0271678X251314682)
Supplement: sj-pdf-1-jcb-10.1177_0271678X251314682 - Supplemental material for Correlation of zero echo time functional MRI with neuronal activity in rats [file sj-pdf-1-jcb-10.1177_0271678X251314682.pdf]

Supplementary Video S1. A recording of air puff whisker stimulation at 20 Hz slowed down 8-fold.

(Separate MP4 file downloadable on JCBFM website.)

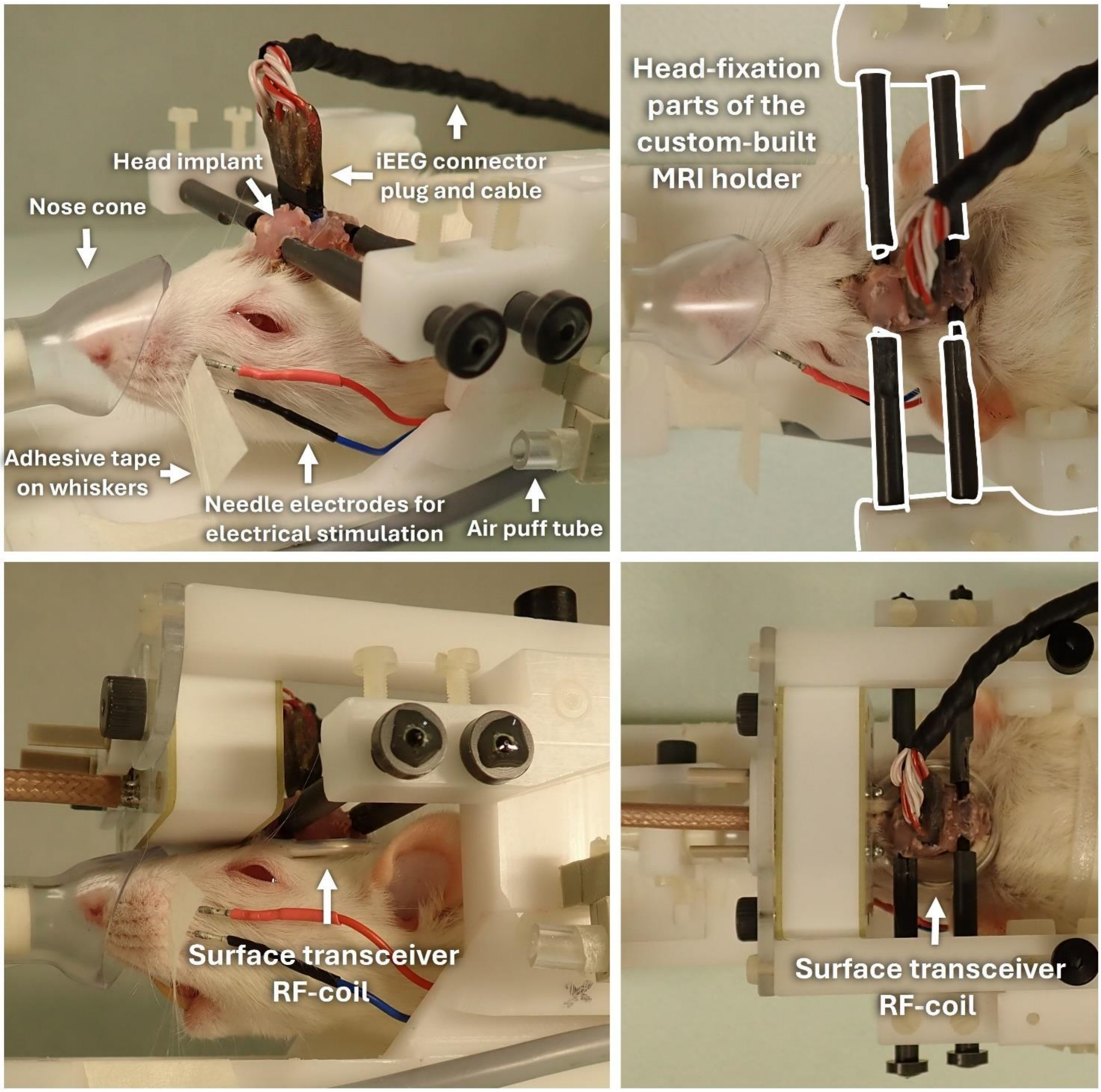

Supplementary Figure S1. The experimental setup. Anesthetized rats were fixed to the custom-built MRI holder via surgically placed head implants. When the rat was in the holder, the iEEG electrodes were connected to the recording system via the connector plug on top of the implant. The electrical stimulation was performed using needles in the whisker pad; the air puff stimulation was performed with pressurized air puffed on a piece of adhesive tape on the whiskers. The upper and lower pair of pictures showcase the setup without and with the RF-coil in place.

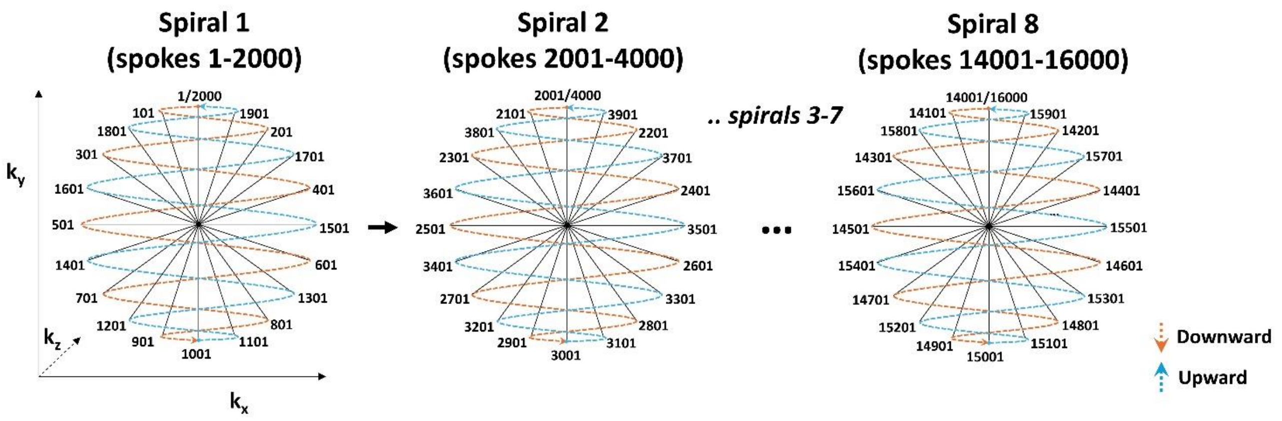

Supplementary Figure S2. The acquisition trajectory in Group 1 fMRI experiments. When performing zero-TE fMRI with the standard one-volume pole-to-pole spiral trajectory, brief pauses in acquisition occur between volumes. When spokes across volumes are combined, such as in EVER-SWIFT,[14] these pauses in acquisition are a source of signal fluctuation and temporal obscurity. Therefore, to avoid these pauses in Group 1 EVER-SWIFT experiments, we used a modified trajectory that allows ~16 s of continuous acquisition. 16000-spoke (8 x same 2000 spokes) volumes were acquired using a spiral trajectory traveling from pole-to-pole and back eight times, with every 2000 spokes repeating the same trajectory with full k-space coverage. For clarity, only every 100th spoke is shown, and the successive downward and upward trajectories are colored differently. Using this 16000-spoke acquisition trajectory, we were able to reconstruct 14-s EVER-SWIFT time courses free of any effects from delays in acquisition (see Supplementary Figure S3).

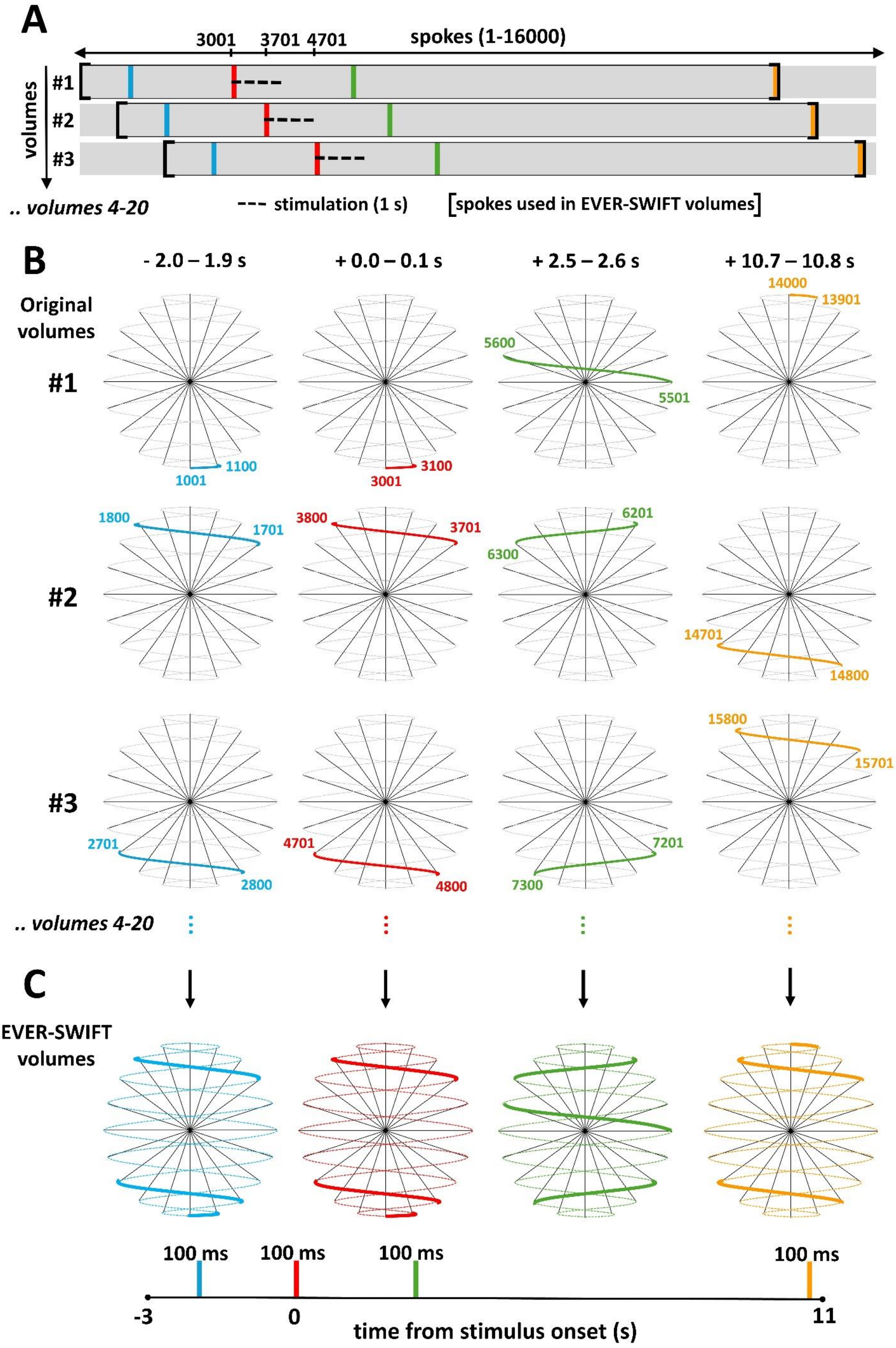

A
3001
3701
4701
spokes (1-16000)
volumes
#1
#2
#3
.. volumes 4-20
--- stimulation (1 s)
[spokes used in EVER-SWIFT volumes]
B
- 2.0 – 1.9 s
+ 0.0 – 0.1 s
+ 2.5 – 2.6 s
+ 10.7 – 10.8 s
Original volumes
#1
1001
1100
3001
3100
5600
5501
14000
13901
#2
1800
1701
3800
3701
6201
6300
14701
14800
#3
2701
2800
4701
4800
7201
7300
15800
15701
.. volumes 4-20
C
EVER-SWIFT volumes
100 ms
100 ms
100 ms
100 ms
-3
0
11
time from stimulus onset (s)

Supplementary Figure S3. Group 1 EVER-SWIFT fMRI experiments. See Supplementary Figure S2 for an explanation of the 16000-spoke volumes. A high pseudo-temporal resolution (~100-ms) was achieved by repeating a ~1-s whisker stimulus 20 times and then retrospectively reorganizing the acquisitions into a single time course.[14] A stimulus was delivered at the start of one of the 100-spoke sections between spokes 3001-5000 in a random order; in **(A)**, the timing of 3 stimuli is shown for illustration. In **(B)**, the portions of k-space covered with the 3 stimuli in 4 different time windows relative to stimulus onset (blue, red, green, yellow) are shown. After 20 stimulus repetitions, the 20 original 16000-spoke volumes were reorganized into 140 2000-spoke volumes, with each volume consisting of acquisitions within the same 100-ms time window relative to stimulus onset**;** in this way, a 14-s fMRI time course with a 100-ms resolution was obtained **(C)**. Note that for each original volume, 2000 spokes were discarded from the start or end of the 16000 spoke acquisition **(A)**. To increase the interval between stimuli, two additional volumes were acquired after each of the stimulus-containing volumes shown here. These volumes allowed ~47-s breaks between stimuli and were not used for image reconstruction.

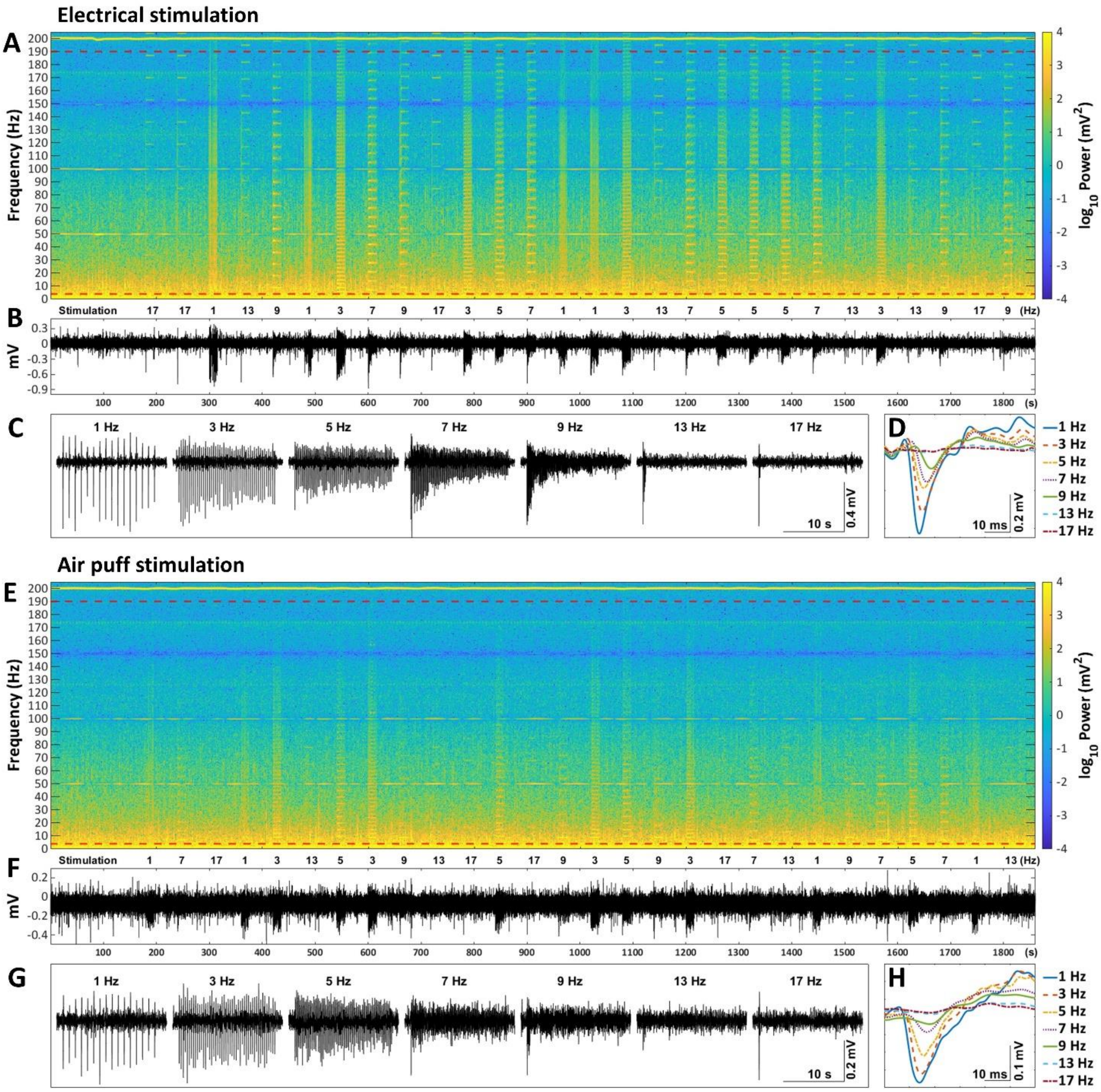

Supplementary Figure S4. iEEG data from one representative fMRI session. Spectrograms for the full 31-min electrical stimulation and air puff stimulation experiments are shown **(A, E)**. Short-time Fourier transform was conducted in 2-s windows with 25% overlap between windows, leading to a temporal resolution of 1.5 s and a frequency resolution of ~0.3 Hz. The red dashed lines mark the borders of the bandpass filter, which were omitted in these spectrograms, but applied in all other iEEG data shown in this work. In **(B)** and **(F)**, the same recordings are shown in the time domain. **(C)** and **(G)** show close-ups of iEEG responses to a train of stimuli, averaged over the 4 blocks of stimuli at each frequency. **(D)** and **(H)** show close-ups of single-stimulus responses, averaged over all stimuli at each frequency. Frequency-adaptation was evident as i) decreases in the amplitude and ii) increases in the latency of consecutive positive and negative voltage deflections.

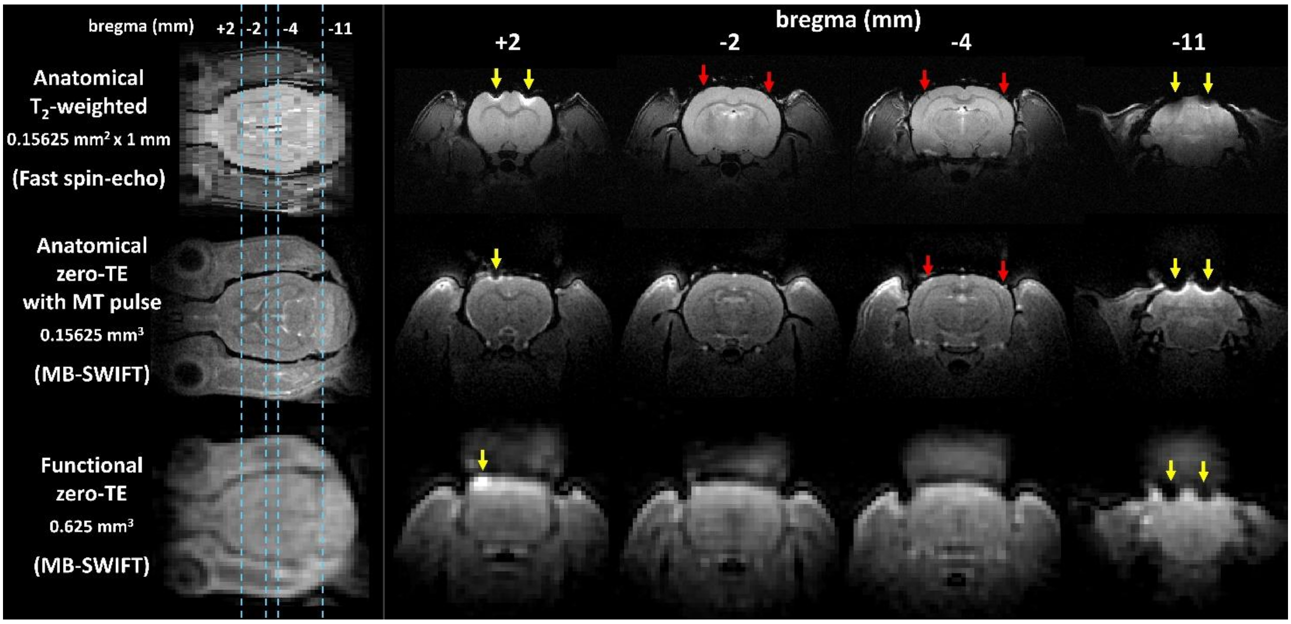

Supplementary Figure S5. Anatomical and functional images from one representative rat. The dashed lines on the horizontal images (left) mark the locations of the coronal slices (right). At -2 mm and -4 mm from bregma, around the coordinates of the tungsten electrodes, only minor bilateral artifacts were visible at the cortical surface in fast spin-echo and some MB-SWIFT images (red arrows). The larger artifacts at +2 and -11 mm from bregma are from the skull screws (yellow arrows). Note that the varying voxel dimensions can affect the representation of the artifacts. The signal above the brain in the functional images is from the head implant (see Supplementary Figure S1). Imaging parameters for fast spin-echo were as follows: TR 2 s, effective TE 30 ms, echo spacing 15 ms, echo train length 2, 128 segments, matrix size 256x256x30, and field-of-view 40x40x30 mm. Abbreviations: magnetization transfer (MT).

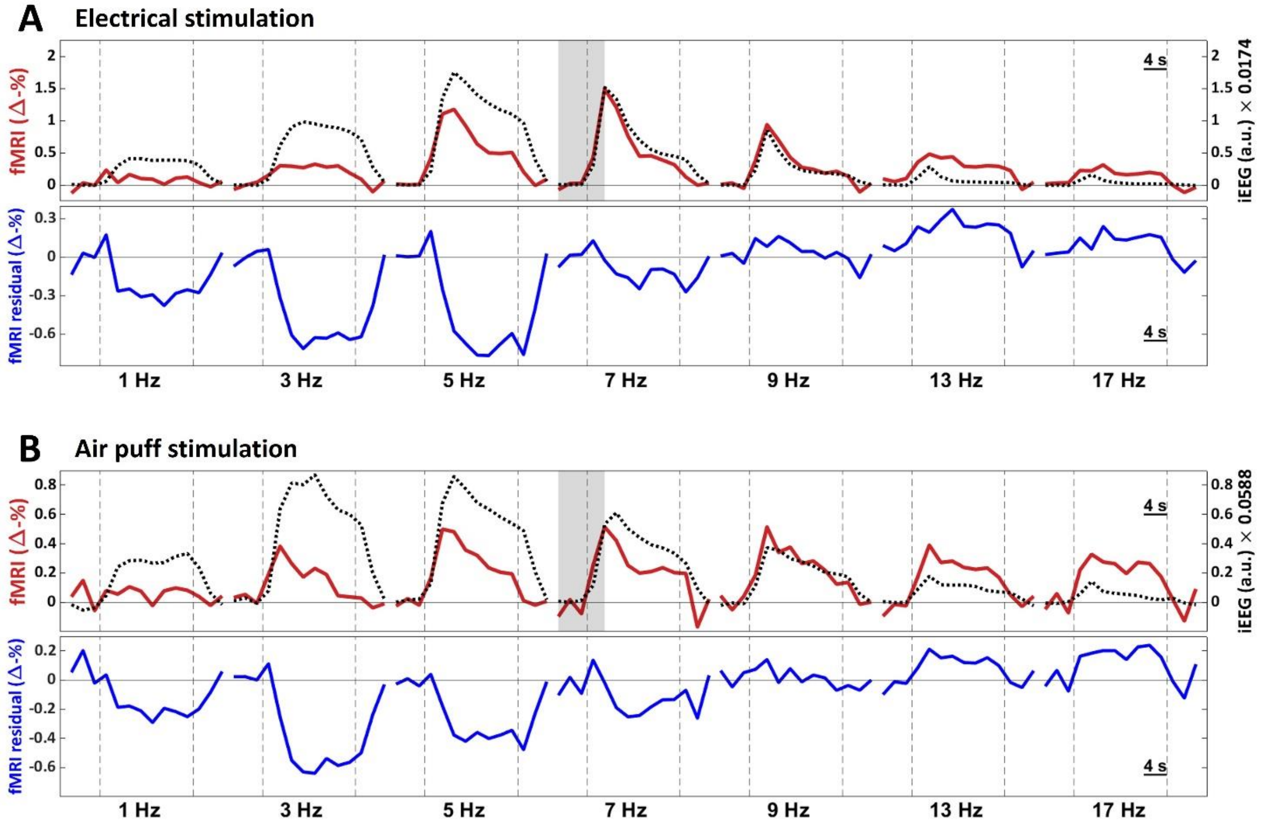

Supplementary Figure S6. Zero-TE fMRI residuals as a function of time and frequency. In contrast to Figure 5, in this figure, the IRF-convolved iEEG time courses have been scaled uniformly across frequencies. The scale was determined by fitting iEEG (dotted black line) to fMRI (red line) at the beginning of the 7 Hz response (shaded window). The regression coefficients were 0.0174 for electrical stimulation **(A)** and 0.0588 for air puff stimulation **(B)**. The differences between the time courses, i.e., the fMRI time course residuals, are shown in the lower panels.
